# Malignant Melanoma Bone Metastasis With Unknown Primary Site: A Case Report and Literature Review

**DOI:** 10.1155/cro/1437495

**Published:** 2026-04-19

**Authors:** Cao Wang, Yan Liu, Lei Han, Xin Qu, Hang Yang, Fengming Ran, Dongqi Li, Dachang Xu, Chenying Yang, Zuozhang Yang

**Affiliations:** ^1^ Bone and Soft Tissue Tumors Research Center of Yunnan Province, Department of Orthopaedics, The Third Affiliated Hospital of Kunming Medical University, Yunnan Cancer Hospital, Peking University Cancer Hospital Yunnan, Kunming, Yunnan, China, ynszlyy.com; ^2^ Department of Rehabilitation Medicine, The Second Affiliated Hospital of Kunming Medical University, Kunming, Yunnan, China, kmmc.cn; ^3^ Department of Pathology, The Third Affiliated Hospital of Kunming Medical University, Yunnan Cancer Hospital, Peking University Cancer Hospital Yunnan, Kunming, Yunnan, China, ynszlyy.com

**Keywords:** bone metastasis, case report, malignant melanoma, unknown primary site

## Abstract

**Introduction:**

Cases of malignant melanoma first identified in bone are relatively uncommon. Approximately 4.3% of the patients will have metastasis, among which 23% will have metastasis to the bone.

**Case Presentation:**

This article presents a case involving malignant melanoma with a metastatic lesion located at the distal femur, which was the initial symptom observed. The patient was a 21‐year‐old male who was admitted to the hospital due to intermittent pain in his lower left thigh. Imaging studies indicated bone destruction in the distal left femur, and pathological and immunohistochemical confirmed metastatic malignant melanoma. The patient underwent “resection of the tumor segment from the left distal femur followed by artificial knee arthroplasty,” after which he received chemotherapy according to established protocols for advanced malignant melanoma. Currently, the patient′s condition is stable, and no recurrence or metastasis has been observed upon re‐examination.

**Discussion:**

This article details the diagnosis, treatment, and prognosis of a patient with malignant melanoma bone metastasis originating from an unknown primary lesion. Additionally, it reviews and analyzes relevant literature concerning malignant melanoma bone metastasis, emphasizing that clinicians should provide timely local and systemic interventions for patients presenting with malignant melanoma bone metastasis, even when the primary lesion remains unidentified.

**Conclusion:**

For patients with malignant melanoma bone metastasis with unknown primary site, after a clear pathological diagnosis, surgical intervention and postoperative systemic treatment should be carried out in a timely manner to improve the overall survival rate of the patients.

## 1. Introduction

Malignant melanoma (MM) is a type of malignant tumor that arises from melanocytes, predominantly affecting the skin and mucous membranes. Advanced MM frequently metastasizes to various sites, including the lungs, liver, brain, and bones. Bone metastases typically occur in the context of widespread metastatic disease; thus, cases of MM presenting with bone metastasis as an initial symptom are relatively uncommon [1, 2]. This article presents a case involving a patient with metastatic MM localized in the distal femur. A comprehensive full‐body examination revealed no primary lesion or additional metastatic foci. Following local surgical intervention and systemic chemotherapy aligned with established treatment protocols for advanced MM, the patient′s condition was effectively managed. Subsequent follow‐up indicated no recurrence or further metastasis of the tumor.

## 2. Case Report

**Figure 1:**
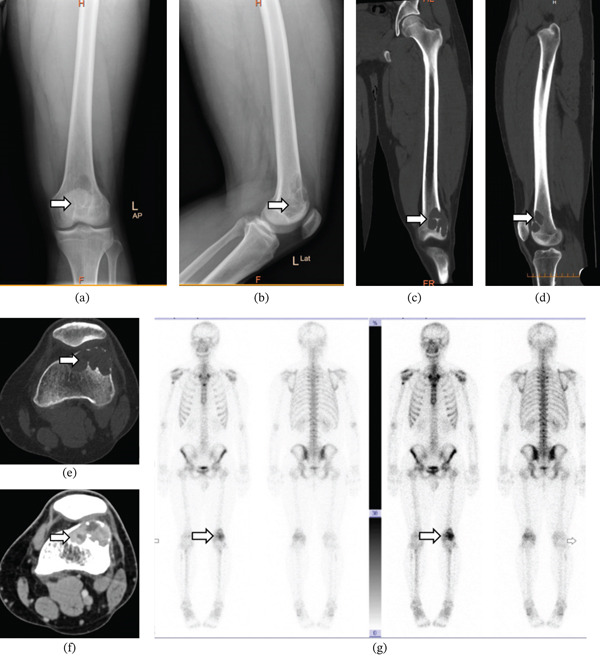
(a–b) Anteroposterior and lateral x‐ray examination of the left femur: Osteolytic bone destruction at the anterior margin of the lower segment. Notable findings include swelling, thinning, and discontinuity of the adjacent cortical bone, as well as roughness observed in the local cortical surface. (c–f) CT imaging of the lower left femur: An expansive, osteolytic area characterized by bone destruction accompanied by a soft tissue mass located at the anterior edge of the lower left femur. Within this region, there is a soft tissue density shadow present; however, no sclerotic margin is observed at the periphery. The boundary of the bone destruction area is distinctly defined, and there are no interruptions or discontinuities noted in the local bone cortex. (g) Whole body bone static imaging: Abnormal metabolic activity observed in the upper segment of the right humerus and at the distal end of the left femur.

A 21‐year‐old male patient presented to our hospital with intermittent pain in the lower part of his left thigh, and the pain symptoms have persisted for 4 months without any special treatment. He has no history of tumors in the past and no family history of hereditary diseases. Upon admission, a comprehensive series of relevant examinations were conducted. The patient reported experiencing intermittent dull pain near the knee joint on the left side, without any local swelling or decreased skin temperature. Imaging studies revealed that anteroposterior and lateral x‐ray examinations of the left femur demonstrated localized bone destruction at the anterior edge of the lower left femur. A CT scan indicated an expansive osteolytic area accompanied by a soft tissue mass at the same location, with well‐defined boundaries. The area of bone destruction is approximately 3.8 × 3.0 cm, and the size of the soft tissue mass is about 3.8 × 5.1 cm. Whole‐body bone imaging results showed abnormal metabolic activity in both the upper segment of the right humerus and at the distal end of the left femur; however, no significant metabolic abnormalities were detected in other skeletal regions (Figure 1). Laboratory test results are follows: ESR (1.00 mm/h), Ca (2.43 mmol/L), CRP (0.85 mg/L), CREA (92.00 *μ*mol/L), and ALP (74.00 U/L).

**Figure 2:**
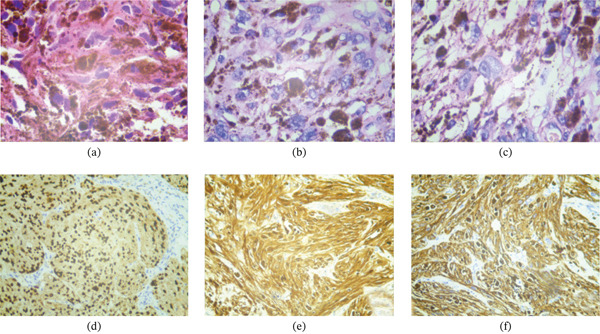
(a–c) Hematoxylin and eosin stain (H&E) (×400): The HE morphology of tumor cells is characterized by their large and atypical appearance, accompanied by a significant presence of melanin granules; (d) SOX10 positive (+); (e) S‐100 positive (+); (f) HMB45 positive (+).

Based on the examination results, there was osteolytic bone destruction in the lower segment of the left femur and the diagnosis was not yet clear. Therefore, under the mobile C‐arm x‐ray machine, a puncture needle was used to enter from the inner side of the lower segment of the left thigh, and the lesion in the lower segment of the left femur was punctured out. The lesion was black, brittle, and a pathological examination was conducted. The immunohistochemical analysis revealed the following results: VIM (+), CK (−), EMA (−), DES (−), SMA (−), CD34 (−), S‐100 (+), SOX10 (+), HMB45 (+), MelanA (partially +), CD68 (−), P63 (−), KI‐67 (+) with approximately 10% positivity (characterized by a significant presence of pigment). Additionally, CYCLIND1 showed focal positivity, whereas P16 and CD163 exhibited partial positivity. Based on both HE staining and immunohistochemical findings, a diagnosis of metastatic MM in the left femur was established as a primary consideration, whereas other pigmentation‐rich tumors were ruled out (Figure 2).

### 2.1. Physical Examination

No black patches or moles were observed on the patient′s entire skin (including the perineum and perianal area), and the conjunctiva of the eyes showed no congestion or pathological changes, the sclera showed no yellowing, the cornea was transparent, there were no opacities, white spots, ulcers, or vascular proliferation, the iris texture was clear, and no pigmented lesions were found in the eyes. The oral mucosa was smooth, without black spots, bleeding, or ulcers, and the pharynx showed no congestion. To further investigate the primary lesion, a PET/CT examination was performed. The results indicated that bone destruction in the lower segment of the left femur accompanied by increased metabolism and thickening of the rectal wall, suggesting a possible malignancy. Furthermore, the PET/CT examination did not reveal any lesions in the right humerus. Based on the PET/CT results, we suspect that it might be the metastasis of rectal MM to the femur and then we performed a colonoscopy on the patient, but the result was proctitis, so this conjecture was overturned.

**Figure 3:**
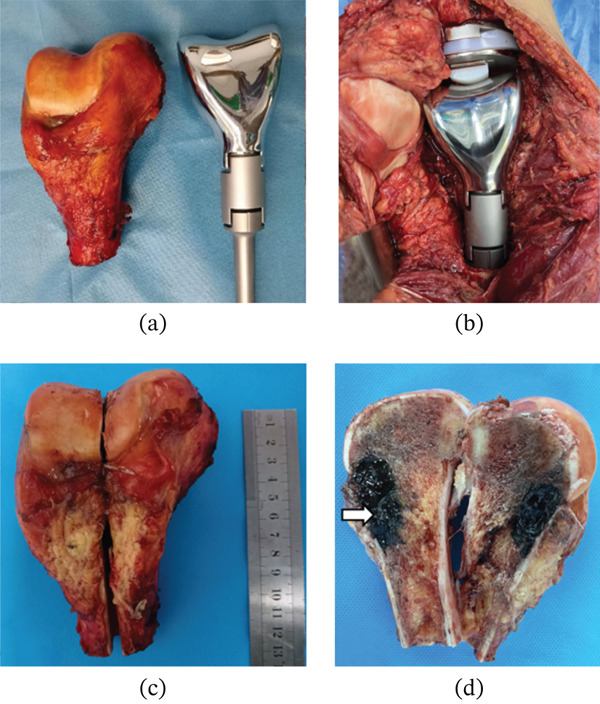
(a–b) Gross specimen of the resection of metastatic malignant melanoma from the left distal femur, along with the artificial knee joint. (c–d) Gross specimen of the resection of metastatic malignant melanoma from the left distal femur, meticulously dissected and examined. Erosive tissue was observed at the distal femur, characterized by a soft texture and black coloration.

Based on the examination results, the patient is considered to have a MM left femur metastasis with unknown primary site. The next treatment plan is surgical intervention. The patient and their family members were informed of the condition and the surgical procedure, and signed consent form was obtained. After completing the preoperative preparations, a “left distal femoral tumor segment resection + artificial knee joint replacement” procedure was performed. During the operation, the left thigh and the inner side of the left knee joint were used as the entry points. The original biopsy channel was removed, and the knee joint capsule was incised; the synovium around the joint was removed, the anterior and posterior cruciate ligaments were severed, the vascular and nerve bundles on the posterior side of the tumor were separated while protecting them, and the lower segment of the femur was cut at a distance of 12 cm from the knee joint surface. The tumor segment was completely removed, and the tissue of the femoral stump was subjected to intraoperative pathological examination. The result was negative. After opening the femoral tumor segment, the lesion was found to be black and brittle (Figure 3). The wound was carefully rinsed, the articular cartilage of the tibial plateau was removed, the tibial epiphysis was retained, the tibia was expanded, a mold was made, the tibial force line was measured, a suitable tibial segment biotype prosthesis was installed, and then the femoral stump was expanded and a suitable length femoral segment prosthesis was installed. Connect and tighten the prosthesis of the femoral segment and the tibial segment. The length was appropriate and the knee joint movement was good. After rewashing the wound, the patellar ligament and the knee joint capsule were reconstructed, and the wound was sutured layer by layer. The surgery was successful. The patient recovered well after the operation, and the range of motion of the left knee joint was good.

**Figure 4:**
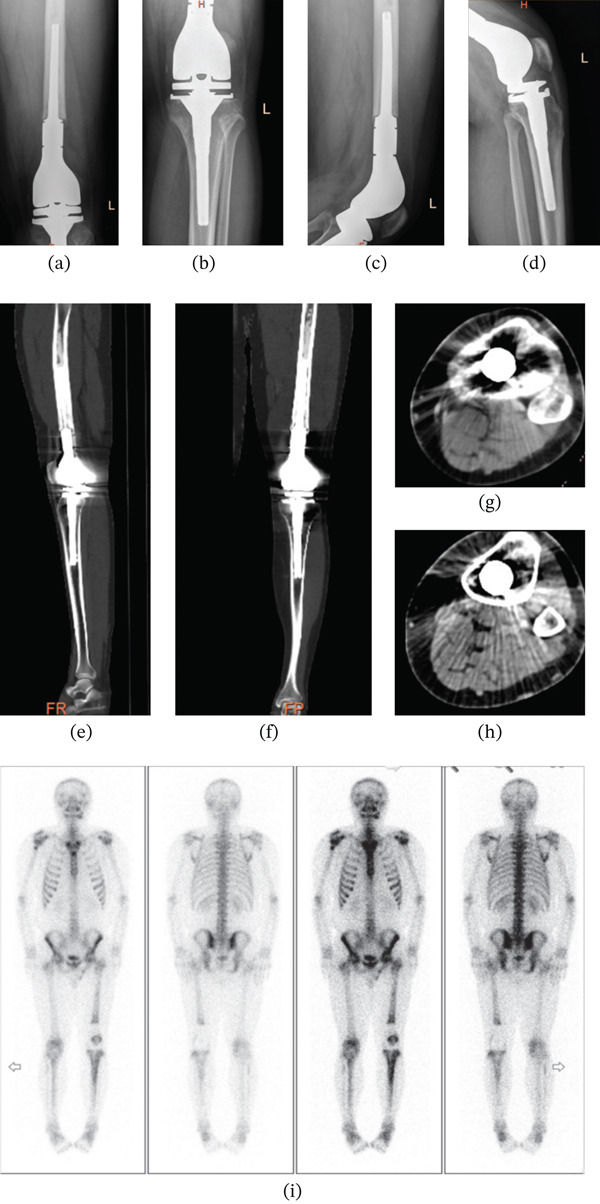
(a–h) DR and CT reexamination following surgery: Observations indicate changes after the absence of the lower left femur and the implantation of an artificial knee joint, which are largely consistent with preoperative findings; (i) postoperative whole‐body bone static imaging reveals a focal abnormal metabolic increase in the upper segment of the original right humerus, similar to previous assessments, suggesting a benign lesion. The reduced metabolic activity at the lower end of the left femur, along with increased abnormal metabolism in both the knee joint and middle to upper segments of the left tibia, exhibited greater stability compared with prior evaluations. These changes posttumor resection and prosthesis implantation were considered in conjunction with the patient′s medical history.

The postoperative pathological examination results indicated a malignant tumor rich in pigments. Based on the results of HE staining and immunohistochemistry, metastatic MM was initially considered. If necessary, multigene tests such as BRAF, KRAS, and KIT could be conducted. In addition, the retained endodontic tissue and soft tissue margins were not observed to contain any clearly malignant tumor tissue under the microscope. The residual tumor grade (R grade): R0. Subsequently, we conducted further molecular testing, and the results showed TP53 (+), BRAF (−), NRAS (−), KIT (−). Based on the test results, it was recommended that the patient undergo immunotherapy combined with chemotherapy. However, due to economic reasons, the patient refused to undergo immunotherapy. The patient began chemotherapy with the “Dacarbazine + Nedaplatin” regimen in January 2023. The chemotherapy cycle was 5 days, with an interval of 3 weeks, and a total of six cycles. Regular reexaminations were conducted during and after the chemotherapy, and enhanced CT examinations of the lungs and left knee were performed every 3 months, and PET/CT and whole‐body bone imaging examinations were conducted every 6 months. The enhanced CT examination results showed that the lower segment of the left femur was absent and had undergone changes after the implantation of an artificial knee joint. The results of multiple reexaminations showed no significant changes. The results of CT and x‐ray examinations did not show any lesions in the right humerus and the whole‐body bone imaging examination results showed increased metabolism in the upper segment of the right humerus, and no significant changes were observed in multiple reexaminations. The PET/CT reexamination results indicated that the patient′s condition was stable and no recurrence or metastasis lesions were found (Figure 4). The patient′s last follow‐up visit was 30 months postoperation and 25 months since completing the last chemotherapy session. There has been no progression of disease, and the pain in the left thigh has significantly improved, the left knee joint functions well, and the quality of life has increased. The MSTS score of the patient′s left knee joint was 28 points, and the LEFS score was 72 points. The patient continues to be monitored through ongoing follow‐up evaluations.

## 3. Discussion

MM is a neoplasm resulting from the malignant transformation of melanocytes located in the skin, mucous membranes, or other organs, characterized by a high degree of malignancy. Approximately 4.3% of patients will experience metastasis [3]. The primary pathways for the metastasis of MM are hematogenous and lymphatic dissemination, which can theoretically affect any region of the body. Approximately 23% of MM metastases occur in bone, a figure significantly lower than that observed for lung and liver metastases. Notably, 80%–90% of bone metastases from MM are found in the axial skeleton, whereas involvement of the limbs is comparatively rare [4–6]. MM bone metastases are frequently first detected through local symptoms and subsequently diagnosed via imaging techniques [7]. In some instances, patients may present with hypercalcemia and lesions in the bone marrow [8]; however, these cases pose diagnostic challenges in clinical practice as they necessitate the exclusion of other malignancies affecting bones as well as multiple myeloma.

A review of the literature indicates that reports of MM bone metastases are relatively scarce, particularly those involving sites such as the clavicle [9], maxilla [10], ribs [11], skull [12], and sphenoid bone [13]. This suggests that the majority of MM bone metastases occur in axial bones, with even fewer cases reported in limb bones [6, 14–16]. Most patients present with localized pain and dysfunction as initial symptoms. Notably, some individuals were not diagnosed with MM during their first visit; rather, this diagnosis was established through subsequent examinations and pathological evaluations. This article presents a case of metastatic MM characterized by local pain as the initial symptom, which occurred in the distal femur. Importantly, no primary lesion was identified upon further examination and treatment.

Bone metastases from MM with unknown primary sites are relatively rare. Several factors may contribute to the inability to identify the primary site of MM, including spontaneous regression of the primary tumor, ectopic melanocytes present in lymph nodes or visceral organs, and potentially mucosal melanoma located in an obscure area [17]. A retrospective study conducted by Tos et al. revealed that over 80% of patients with metastatic MM of unknown origin remained undetected despite extensive examinations [18]. The findings indicated that patients with metastatic MM of unknown origin exhibited a better prognosis and higher overall survival rates compared with those with metastatic primary cutaneous MM [19, 20].

Pathology remains the gold standard for diagnosing bone metastases of MM. Melanoma often exhibits various cytological morphologies, and conventional staining techniques can complicate the differentiation between tumors with similar histological origins, leading to diagnostic challenges. Immunohistochemical methods have proven effective in distinguishing tumor cells based on their morphology and origin, thus becoming an essential adjunctive examination for melanoma diagnosis. Currently, SOX10, S‐100, HMB‐45, and Melan A are recognized as classic immunohistochemical staining markers [21–23]. The immunohistochemical findings in this case demonstrated that SOX10(+), S‐100(+), HMB‐45(+), and Melan A(+) were consistent with a diagnosis of MM.

MM that has metastasized to the bone typically presents with osteolytic changes. Clinically, it needs to be differentiated from many other diseases that cause osteolytic bone destruction. From a histological perspective, metastatic bone tumors generally have the cellular morphology characteristics of the primary tumor. Osteosarcoma tumor cells directly produce immature bone‐like tissue or bone, and the cells exhibit high atypia and pleomorphism [24, 25]. There are osteoclast‐like giant cell components in osteoclastoma [26]. In addition, metastatic MM can exhibit a variety of cellular morphological characteristics, and it needs to be differentiated from poorly differentiated carcinomas, sarcomas, and hematological malignancies [27, 28]. At this time, pathological and immunohistochemical methods can be employed. Primary clear cell sarcoma of bone is relatively rare in clinical practice. Its immunological characteristics are highly consistent with those of MM, and it specifically expresses melanocyte markers (HMB‐45, S‐100, and MelanA). It needs to be differentiated from MM bone metastasis. The distinguishing point lies in the presence of a t(12; 22), (q13; q12) chromosomal translocation in primary clear cell sarcoma of bone, which leads to the fusion of oncogene ATF1 and EWSR1, generating the EWSR1‐ATF1 fusion protein [29–31](Table 1).

**Table 1:** Differential diagnosis of malignant melanoma bone metastasis from several other diseases.

	Imaging findings	Cellular morphological characteristics	Immunological characteristics	Molecular testing
Malignant melanoma bone metastasis	Osteolytic bone destruction; soft tissue tumor.	Large in size; unusual shape; and rich melanin granules.	HMB‐45(+); S‐100(+); MelanA(+).	BRAF(+); NRAS(+); KIT(+).
Metastasis from other primary tumors	Bone destruction (osteolytic, osteogenic, or mixed)	Morphology of the primary tumor cells.	—	—
Osteosarcoma	Bone cortical destruction (Codman triangle); soft tissue tumor.	Immature bone‐like tissue; high atypia and pleomorphism.	SATB2(+); osteocalcin(+).	—
Giant cell tumor of bone	Asymmetric bone destruction; “soap‐bubble” appearance.	Osteoclast‐like giant cell; mononuclear stromal cells; and multinucleated giant cells	H3F3A(+); CD68(+); RANK/RANKL(+).	H3F3A mutation.
Primary clear cell sarcoma of bone	Osteolytic bone destruction; thinning of the bone cortex.	Nests of spindle tumor cells; clear cytoplasm.	HMB‐45(+); S‐100(+); MelanA(+).	t(12; 22), (q13; q12) chromosomal translocation.

The median survival for patients with metastatic MM is reported to be only 6–9 months. A study by Conway et al. indicated that the overall survival of patients with bone metastasis from MM is shorter compared with those with lung metastasis and lymph node metastasis [32]. The most common treatment approach for these patients involves palliative systemic therapy, such as dacarbazine monotherapy [33, 34]. Although combined cytotoxic chemotherapy or immunotherapy has been shown to be more effective in reducing tumor volume than monotherapy, there is no significant difference in overall survival rates; moreover, combination therapies tend to have more side effects. Therefore, clinical treatment should be tailored based on the individual patient′s circumstances. Combination chemotherapy may be particularly suitable for cases requiring rapid reduction of tumor volume due to complications such as tumor rupture, bleeding, or infection [3, 35]. MM bone metastases are often managed through systemic therapy; however, surgical interventions can alleviate pain and enhance local function, thereby improving the quality of life for patients. Reipond et al. documented a case involving a patient with isolated bone metastasis in the distal right radius following surgery for MM in the right thigh; this patient experienced significant improvement in arm function after undergoing active surgical treatment [36]. Tozum et al. performed a total collarotomy on a patient presenting with isolated collarbone metastasis from melanoma [9], whereas Lombardi et al. conducted lesion resection on a patient with isolated MM of the maxilla [10]; both cases demonstrated favorable postoperative prognoses. Surgery remains the primary treatment modality for melanoma of unknown primary (MUP), and surgical resection of MUP metastases has been associated with improved survival rates [37, 38]. Furthermore, postoperative adjuvant systemic palliative care further enhances overall survival outcomes [18].

## 4. Conclusion

To summarize, cases of limb bone metastasis originating from MM are relatively uncommon, particularly in instances where the primary lesion remains unidentified. These cases pose significant challenges for clinical diagnosis. Upon admission, it is essential to conduct relevant examinations for such patients and perform local lesion sampling through biopsy. Following a definitive pathological diagnosis, timely surgical intervention and postoperative systemic treatment should be implemented to enhance the functionality of the affected limb and improve the overall survival rate of the patient. Given that the primary lesion is often unidentifiable in most cases, extensive tumor screening is deemed unnecessary. It is recommended that for these patients, alongside histopathological examination and PET/CT imaging, a thorough medical history inquiry and standard physical examination be conducted as well.

## Author Contributions

C.W. and Y.L.: data interpretation, writing the paper, literature review; L.H., X.Q., and H.Y.: manuscript editing and revision; F.R.: collect pathological images and analysis results; D.L., D.X., and C.Y.: clinical data collection and analysis; Z.Y.: formulation or evolution of overarching research goals and aims. C.W. and Y.L. contributed equally to this work and should be considered as co‐first authors.

## Funding

This study was supported by the National Science Foundation of China (82260590); Yunnan Fundamental Research Projects (202401AT070353); Scientific Research Fund project of Education Department of Yunnan Province (2025J0181).

## Ethics Statement

The authors are accountable for all aspects of the work in ensuring that questions related to the accuracy or integrity of any part of the work are appropriately investigated and resolved. Written informed consent was obtained from the patient for publication of this case report and any accompanying images. A copy of the molecular genetic test report is available for review by the editor in chief of this journal on request.

## Conflicts of Interest

The authors declare no conflicts of interest.

## Data Availability

The data that support the findings of this study are available from the corresponding author upon reasonable request.
